# Supplementary material for: Physicochemical Evidence that Francisella FupA and FupB Proteins Are Porins
Source: Int J Mol Sci. 2020 Jul 31;21(15):5496. doi: 10.3390/ijms21155496 (PMC7432831; doi:10.3390/ijms21155496)
Supplement: Supplementary file 1 [file ijms-21-05496-s001.pdf]

Table S1. List of used primers.

Name	Gene	Primer Sequences (5′-3′)
FupA/B_ DeltaN_LVS	*FTL_0439*	FupA -88F<br>**GGGGACAAGTTTGTACAAAAAAGCAGGCT**TA<u>GAAAACCTGTACTTCCAGGGT</u>gttgtttcacaagggggccLVSFupA _0439Rev **GGGGACCACTTTGTACAAGAAAGCTGGGT**C<u>TTATTA</u>GATATAAACTGAAAGATCTAATG
FupA_DeltaN_Fno	*FTN_0444*	FupA -88F FnoFupA _0444Rev<br>**GGGGACCACTTTGTACAAGAAAGCTGGGT**C<u>TTATTA</u>TACGTATACCGACATATCCAGAG
FupB_DeltaN_Fno	*FTN_0445*	FnoFupB_78F<br>**GGGGACAAGTTTGTACAAAAAAGCAGGCT**TA<u>GAAAACCTGTACTTCCAGGGT</u>gttaataactctcaacaattagatgc<br>FnoFupB_0445Rev<br>**GGGGACCACTTTGTACAAGAAAGCTGGGT**C<u>TTATTA</u>aatataaactgaaagatctagtg

Primers were designed to contain the universal *att*B1 and *att*B2 adapter sequences (in bold). The forward primers also contained a TEV protease site allowing removal of the tags while the reverse primers contained two consecutive stop codons (underlined).
